# Editorial: Phonological and Phonetic Competence: Between Grammar, Signal Processing, and Neural Activity

**DOI:** 10.3389/fpsyg.2015.01899

**Published:** 2015-12-23

**Authors:** Ulrike Domahs, Hubert Truckenbrodt, Richard Wiese

**Affiliations:** ^1^Faculty of Education, Free University of Bozen-BolzanoBolzano, Italy; ^2^Zentrum für Allgemeine SprachwissenschaftBerlin, Germany; ^3^Institut für Germanistische Sprachwissenschaft, University of MarburgMarburg, Germany

**Keywords:** prosody, phonetics, phonology, speech perception, speech production, language development, language change

## Introduction

The present collection addresses the place and role of phonology (as an object of study, not as a scientific field) within a wider range of neighboring domains. Generally, the relevance of phonological structure in language may be claimed to derive from the fact that phonology constitutes a domain of its own within language (along with syntax, semantics, morphology), but also interfaces intimately with other domains such as cognition, articulation, and perception in general. From this dual nature, it follows that phonology may be an object of linguistic description and theory (for an overview see Goldsmith, 1995; de Lacy, 2012) as well as an object of cognitive and behavioral studies (for an overview see Cohn et al., 2012). Ideally, however, theoretical and empirical studies keep this dual nature of phonology in mind and pay attention to both sides of the coin.

Articles in the present Research Topic attempt to capture different aspects of this overall discussion. The starting point for this Research Topic was a Priority Programme on experimental research in phonology and phonetics funded by the German Science Foundation (DFG; SPP 1234). Based on this programme, the aim of this Research Topic is to draw together empirical work in the field of segmental and prosodic processing and representation and phonological theory.

Contributions address the interface of the speech sound systems investigated in phonology, the representations of articulated speech, perception, acquisition and processing established in phonetics, psycholinguistics, and neurolinguistics. Main topics of investigation include: (1) sounds and sound-changing processes—systemic and functional aspects, (2) prosodic units such as syllables and metrical feet—systemic properties, processing, and phonetic consequences, and (3) tones as building blocks of the sentence melody—their relation to the level of linguistic expressions on the one hand, their phonetic realization (e.g., tonal height and contours) and perception on the other hand. In addition, topics (1) and (2) extend to the question how phonological representations are stored in the mental lexicon: specified minimally in terms of categorical phonological information or as variable phonetic imprint of the exemplars in the input.

Diagonally to these thematic domains, the present Research Topic shows a strong focus on up-to-date experimental methods. Contributions go far beyond traditional linguistic analysis, and make use of psycho- and neuro-linguistic methods.

## The contributions

Sound and sound-changing processes are investigated by Bukmaier and colleagues, Schild and colleagues, Truckenbrodt and colleagues, van der Vijver and Baer, Poellmann and colleagues, and Zimmerer and Reetz. Bukmaier and colleagues present production and perception experiments that provide evidence for a process of sound change in which the neutralization of /s/ and /∫/ to /∫/ before stops in Augsburg German is influenced by the Standard German contrast between /s/ and /∫/. The continuous function of the exposition to Standard German supports models that adhere to the exemplar theory of speech. Changes in sound perception during early childhood were studied by Schild and colleagues. In an ERP study with pre-schoolers, beginning readers, and adults, the authors investigated how stressed and unstressed syllables prime German word targets when prime and target overlap in phonemes and stress patterns. Age-related differences show that the processing of phonemes, but not the processing of stress is modulated by literacy acquisition.

An MMN-study devoted to investigate whether pre-attentive processing is sensitive to a syllable-related phonological process of German, namely final devoicing, was conducted by Truckenbrodt and colleagues. The authors found MMN effects for deviants violating final devoicing showing that even early pre-attentive auditory processing is modulated by syllable-related and automatic lexical phenomena. Final devoicing as the cause of voicing alternations in singular–plural pairs is also in the focus of the contribution by van de Vijver and Baer-Henney. In their production study, 5 and 7 years old children and adults produced plural forms out of pseudowords that required either voicing or vowel alternations. Age-related decrease of voicing and increase of vowel alternations show that generalizations are lexicon-based and rely on the frequencies of certain processes that vary between child and adult lexicon.

More indirectly connected to the topic of sound changing processes are two contributions on the production and perception of reduced forms displaying either sound deletions or reductions of phonological features. Poellmann and colleagues performed a series of eye-tracking experiments on the perception of reduced forms in which segments were either reduced or deleted. The experience with inconsistent pronunciations leads to a greater perceptual flexibility in dealing with other forms of reduction than does the experience with consistent pronunciations. The processing of reduced forms is also investigated by Zimmerer and Reetz. More specifically, they were interested in the sensitivity to compensatory acoustic cues left when a final /t/ is deleted, and investigated whether German listeners are able to reconstruct a final /t/ when confronted with reduced forms. They found that /t/ was reconstructed in only 45% of items presented. This finding is discussed in the light of the experimental methodology and stimuli used and the acoustic cues indexing final /t/ deletion in German.

The role of prosodic entities and/or their representation is investigated by Bien and colleagues, Samlowski and colleagues, Domahs and colleagues, Domahs and colleagues, Häuser and Domahs, Heisterueber and colleagues, and also Schild and colleagues. In an ERP study using a word fragment priming paradigm, Bien and colleagues found effects that underpin the relevance of the syllable for language processing and lexical access. Samlowski and colleagues investigated the role of a number of prosodic and grammatical factors for syllable pronunciation in German. Some of these factors (word stress and sentence boundaries, lexical classes) were demonstrated to influence phonetic details (especially duration) of syllables corresponding to prefixes and function words.

How syllables are parsed into feet and whether feet are constructed beginning from the right or left edge of words has been investigated by Domahs and colleagues. The selection of the antepenultimate or final syllable as syllable bearing main stress in trisyllabic pseudowords is found to correlate with the working memory capacity of participants in a pseudoword production task.

A study on foot properties is presented by Domahs and colleagues. Their EEG results support evidence for bimoraic trochaic feet as processing units in the word stress system of Cairene Arabic. In addition, prosodic structure in Cairene Arabic is shown to be generated and constructed actively in online processing. The highly predictable word stress system does not lead to limitations in the sensitivity to word stress, i.e., there is no stress-deafness as predicted, among others, by Peperkamp and Dupoux (2002).

Regarding the question where lexical stress representations are functionally localized, Häuser and Domahs reviewed a series of published patient studies: all patients with a representational deficit in word stress processing had lesions in their language-dominant hemisphere. Word stress processing relies mainly on the functioning of the left hemisphere. However, Heisterueber and colleagues show that stress processing is also subject to inter-individual differences, as shown in an fMRI-study performed with German native speakers who participated in a sequence recall task testing the capacity to represent segmental and suprasegmental information on an abstract level. The authors report inter-individual differences in behavioral and neural activation patterns for word stress processing modulated by individual auditory processing and working memory capacities.

Finally, Kügler and Gollrad presented production and perception studies on contrastive meaning components of a rise-fall contour in German: a pitch accent carrying a particular meaning has a preference to occur with a context that triggers this particular meaning. Their findings suggest that the alignment and scaling of the accentual peak are sufficient to license a contrastive interpretation of the nuclear rise-fall contour.

## Author contributions

UD, HT, and RW conceived the topic of this Research Topic. All authors participated in the editorial work.

### Conflict of interest statement

The authors declare that the research was conducted in the absence of any commercial or financial relationships that could be construed as a potential conflict of interest.
